# IgG Placental Transfer in Healthy and Pathological Pregnancies

**DOI:** 10.1155/2012/985646

**Published:** 2011-10-01

**Authors:** Patricia Palmeira, Camila Quinello, Ana Lúcia Silveira-Lessa, Cláudia Augusta Zago, Magda Carneiro-Sampaio

**Affiliations:** ^1^Departamento de Pediatria, Faculdade de Medicina, Universidade de São Paulo, Av. Dr. Enéas Carvalho de Aguiar, 647, Cerqueira César, São Paulo, SP 05403-900, Brazil; ^2^Laboratório de Investigação Médica (LIM-36), Instituto da Criança, Hospital das Clínicas, Av. Dr. Enéas Carvalho de Aguiar, 647, Cerqueira César, São Paulo, SP 05403-900, Brazil; ^3^Departamento de Parasitologia, Instituto de Ciências Biomédicas, Universidade de São Paulo, Av. Prof. Lineu Prestes, 1374, Cidade Universitária, São Paulo, SP 05508-000, Brazil

## Abstract

Placental transfer of maternal IgG antibodies to the fetus is an important mechanism that provides protection to the infant while his/her humoral response is inefficient. IgG is the only antibody class that significantly crosses the human placenta. This crossing is mediated by FcRn expressed on syncytiotrophoblast cells. There is evidence that IgG transfer depends on the following: (i) maternal levels of total IgG and specific antibodies, (ii) gestational age, (iii) placental integrity, (iv) IgG subclass, and (v) nature of antigen, being more intense for thymus-dependent ones. These features represent the basis for maternal immunization strategies aimed at protecting newborns against neonatal and infantile infectious diseases. In some situations, such as mothers with primary immunodeficiencies, exogenous IgG acquired by intravenous immunoglobulin therapy crosses the placenta in similar patterns to endogenous immunoglobulins and may also protect the offspring from infections in early life. Inversely, harmful autoantibodies may cross the placenta and cause transitory autoimmune disease in the neonate.

## 1. Introduction

Anti-infectious fetal protection is provided by several factors acting together. The uterine cavity contains innate immune detection and effector systems that maintain sterility, detect infection and, under conditions of substantial microbial invasion, induce expression of mediators that could accelerate lung maturation and induce a preterm labor to deliver the fetus from a threatening environment [1]. The vaginal tract, which is normally colonized with multiple microorganisms, is separated from the normally sterile intrauterine compartment by the cervical plug, which contains several antimicrobial proteins and peptides (APPs), including lactoferrin and *α*-defensins. Inside the uterine cavity, the amniotic fluid contains acute phase proteins, such as soluble CD14 and lipopolysaccharide- (LPS-) binding protein (LBP), which modulates the endotoxic activity of LPS and cationic membrane-active APPs, such as lactoferrin, bactericidal/permeability-increasing protein, histones, and defensins [2]. In preterm labor, increased concentrations of group II phospholipase A2 are found, and this enzyme has been associated with a remarkable potency against Gram-positive bacteria [3, 4].

At birth, the neonate presents an increased susceptibility to infectious agents due to functional immaturity of his/her immune system. Some functions are particularly immature, whereas other aspects are functional at birth even in extremely preterm newborn infants. Neutrophils have a small storage pool at birth, and this cell lineage is less responsive to chemoattractants than later in development. Monocytes/macrophages are reported to be functionally adequate but have limitations in chemotactic responsiveness. Infant blood monocytes produce less IFN-*α*, IFN-*γ*, and IL-12 subunit p70 (IL-12 p70) than cells obtained from adults. However, production of these cytokines rapidly increases between birth and 1 or 2 years of age. In contrast, infant cells show a greater capacity to produce IL-10 and to induce IL-17-producing helper T cells (Th17 cells) in response to Toll-like receptor (TLR) stimulation by producing IL-6 and IL-23 [2]. Furthermore, individual infant cells are less able than adult cells to produce multiple cytokines simultaneously in response to TLR agonists; that is, infant cells are less polyfunctional [5]. The predominance of a Th17-like pattern combined with considerable IL-10 production may contribute to diminished T helper type 1 (Th1) responses, resulting in greater susceptibility to intracellular infections and diminished vaccine responses during infancy [6].

Neonatal T CD4^+^ cells present an intrinsic immaturity with a diminished capacity to generate memory cells and reduced Th1 effector functions such as the production of less IFN-*γ* and lower CD40L expression. These deficiencies seem mainly to be related to the fact that the cells are still naive, having met few antigens [7]. Thymic recent emigrants (TRECs), which are T cells recently migrated from the thymus, are present in a large proportion in the periphery of human infants, and these TRECs are impaired in their acquisition of Th1 function [8]. CD4^+^ T cell responses, but not CD8^+^ T cell responses, develop more slowly in infants than in adults after primary infection with cytomegalovirus or herpes simplex virus [9]. In addition, responses to some vaccines, such as vaccines for hepatitis B virus and oral poliovirus vaccine, result in less Th1 activity and a bias toward Th2 function [10]. The ability of proinflammatory cytokines to induce spontaneous abortion is likely to be an important reason for the strong bias of the maternal and fetal immune systems of multiple mammalian species towards Th2-cell-polarizing cytokines [1, 11]. The Th2 locus is hypomethylated in both human and mouse infants, contributing to the expression of these cytokine genes, which corresponds to the propensity for Th2-polarizing cytokine responses in infants [12, 13]. Thus, infants have a dominant anti-inflammatory cytokine profile that seems to be induced during fetal life [7]. It has been demonstrated that in the in utero environment, CD4^+^CD25^hi^Foxp3^+^ regulatory T cells dominate the fetal circulation, suppressing reactivity to noninherited maternal antigens [14] and possibly promoting a generally suppressive environment. 

Regarding neonatal antibody responses, several studies have shown a delayed onset, lower peak levels, a shorter duration, differences in the distribution of IgG isotypes (with infants showing lower IgG2 than adults), and lower affinity and reduced heterogeneity. Antibody responses to thymus-independent type 2 antigens (including bacterial polysaccharides) are also deficient [15]. There is no transplacental transfer of complement system elements, and neonates have relatively low levels of some components [16]. Furthermore, neonatal and infantile B cells have low expression of CD21 (complement receptor 2), which explains the inadequate response to polysaccharides [17]. Interestingly, the increase in CD21 levels that occurs during development coincides with the response to polysaccharides [18]. 

Considering that after exposure to each new microbe it takes time to develop each specific protective immune response, the placental transfer of maternal immunoglobulins to the fetus is a specific adaptative mechanism that, to some extent, minimizes the deficiencies in antibody production and confers short-term passive immunity. Moreover, additional immune response support is given by the mother through breast milk, which contains functional nutrients and IgA antibodies that provide efficient protection directly after birth by preventing adherence of infectious agents on the mucosal membranes and ultimately their entrance into tissues.

## 2. IgG Placental Transfer Is Mediated by FcRn

In humans, substances that pass from maternal blood to fetal blood must traverse the histological barrier, which consists of two cell layers: the multinucleated syncytiotrophoblasts (STBs) and endothelial cells of the fetal capillaries. Furthermore, fibroblasts and Hofbauer cells (i.e., placental macrophages) are found in the villous stroma and are presumably involved in the binding and trapping of immune complexes [19].

Although this barrier separates the blood in maternal and fetal circulation, it is not a simple physical barrier. A wide range of substances, including nutrients and solutes, are efficiently transferred actively or passively through the placenta to the fetus, and this mechanism is essential for normal fetal growth and development. Most low molecular mass compounds (<500 Da) simply diffuse through the placental tissue interposed between the maternal and fetal circulation. Some low molecular weight substances, such as ions and amino acids, show unidirectional transfer across the placenta. Substances of very high molecular weight do not usually traverse the placenta, but there are a few exceptions, such as immunoglobulin G (IgG), which has a molecular mass of approximately 160 kDa.

Of the five antibody classes, only significant amounts of IgG are transferred across the placenta. On the basis of the observation that whole IgG molecules or Fc fragments of IgG pass into the fetal circulation more readily than F(ab′)^2^ fragments [20], it was hypothesized that IgG Fc receptors (Fc*γ*Rs) on placental cells may be involved in IgG transfer across placenta. Later, it was established that this specific transport of IgG is carried out by the neonatal Fc receptor (FcRn) [21, 22]. This has been demonstrated unequivocally in *ex vivo* perfused placenta by comparing the transport of a recombinant, humanized IgG1 antibody with that of a mutated variant that does not bind to FcRn [23]. FcRn is composed of an integral membrane glycoprotein with an apparent molecular weight of 40–45 kDa for the *α*-chain, which is noncovalently associated with *β*2-microglobulin (*β*2 m) [24]. Thus, while the major ligands of FcRn are IgG and albumin, FcRn is most closely structurally related to major histocompatibility complex (MHC) class I molecules, with which it shares 22%–29% sequence homology. In contrast to other Fc*γ*-receptors, FcRn exhibits a characteristic pH-dependency of IgG binding, demonstrating a high affinity for IgG at pH 6.0, but 100-times lower affinity at physiological pH (7.4) [25]. Thus, FcRn is unable to bind IgG at the apical side of STB facing the maternal blood. 

**Figure 1:**
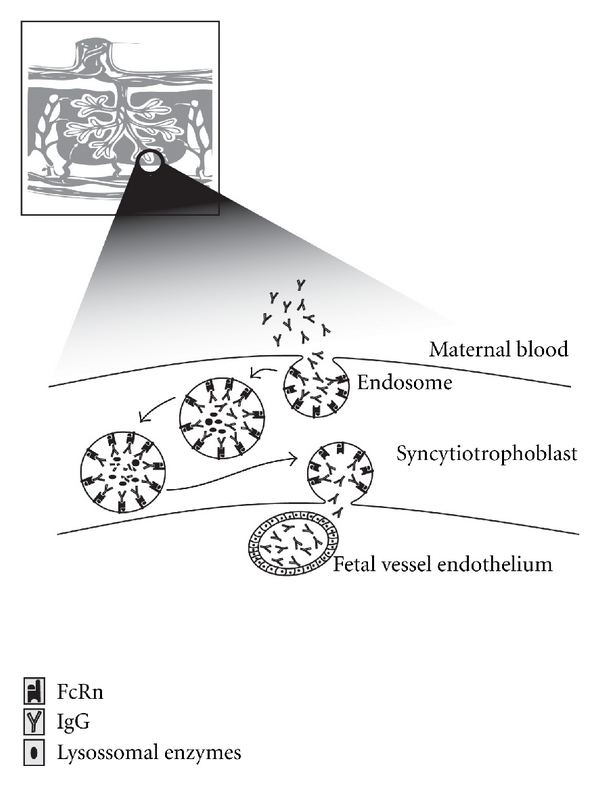
IgG transfer from the mother to the fetus occurs during pregnancy across the syncytiotrophoblasts of the placenta. Syncytiotrophoblasts are bathed in maternal blood and internalize maternal IgG in endosomes. FcRn is expressed on the internal surfaces of the endosome. Upon acidification in the endosome, maternal IgG bound to FcRn is protected from degradation by lysosomal enzymes and then is transcytosed. The endosomes fuse with the membrane on the fetal side of the syncytiotrophoblast, where the physiological pH promotes the dissociation of IgG from FcRn to the fetal circulation. High levels of IgG antibodies cause IgG degradation due to the saturation of FcRn receptors.

It is, therefore, assumed that IgG present in high concentrations in the maternal circulation (10–20 mg/mL) is taken up by fluid-phase endocytosis by STB and then binds to the FcRn in the acidic environment of endosomes [26]. Bound IgG may then be transcytosed to the basolateral side, where it is released upon exposure to neutral pH (7.4). The FcRn molecule may then be recycled to the maternal membrane to perform additional rounds of transcytosis, as observed in other systems [27]. Therefore, pH-dependent binding of IgG to FcRn allows for IgG transport through a cell layer and down a concentration gradient of IgG [28–30] (Figure 1).

Transcytosed IgG may or may not pass through the stroma before reaching the fetal blood vessels. It remains controversial as to whether FcRn is also expressed in the fetal vessel endothelium, where greater evidence exists for the action of alternative Fc receptors in further movement of IgG [31, 32]. This IgG transport model is supported by the *in situ* localization of IgG and FcRn subunits and by studies investigating IgG transport in *ex vivo* perfused placentae [33]. The FcRn *α*-chain has been found to be localized mainly in intracellular vesicles and to a minor extent at the apical membrane of STB of first trimester and term placentae [26, 33, 34].

The function of FcRn also extends to many other sites within the body, where it plays an important role in modulating lifelong humoral and cell-mediated immune responses. It is also expressed in both endothelial and bone marrow-derived cells and plays an integral role in protecting IgG from catabolism, which allows IgG to be recycled to the cell surface and back into the bloodstream, extending its half-life in the serum of adults [35]. FcRn is also expressed in many other tissues in the adult animal, including barrier sites such as the blood-brain interface, the glomerular filter in the kidneys and the intestinal epithelium, where its function of modulating IgG transport to promote host defense or to control immune-complex deposition is still speculative [36].

To be transferred through human placenta, maternal IgG must cross the STBs, the stroma of the intravillous space, and the fetal vessel endothelium. These tissues express unique patterns of various types of Fc receptors of IgG including Fc*γ*RI, Fc*γ*RII, and Fc*γ*RIII. In the placenta, Fc*γ*RI has been found in the loose connective tissue, mononuclear phagocytes, and the Hofbauer cells, which are morphologically defined as macrophages due to their ability to perform phagocytosis and to interact with IgG. Trophoblast cells in term placentae express both Fc*γ*RIII and FcRn. Placental Fc*γ*RIII is a membrane-spanning Fc*γ*RIIIa isoform, which is predominantly expressed by Natural Killer (NK) cells. The binding of Fc*γ*RIII (also called CD16) on NK cells to immune complexes or IgG on target cells, or treatment with an anti-CD16 monoclonal antibody to crosslink membrane spanning Fc*γ*RIII induces NK cell activation. This activation leads to upregulation of the transcripts for cytokines such as IFN-*γ* and TNF-*α* [37, 38]. These observations indicate that Fc*γ*RIIIa on trophoblasts may bind immune complexes or antibody-coated particles in the maternal circulation and may induce the transcription of cytokines or trigger cell-mediated immunity.

Fetal endothelial cells in placenta express Fc*γ*RII and FcRn although data regarding FcRn expression in the endothelium are still conflicting [39].

## 3. Placental Transport of IgG Depends on Maternal Levels

**Figure 2:**
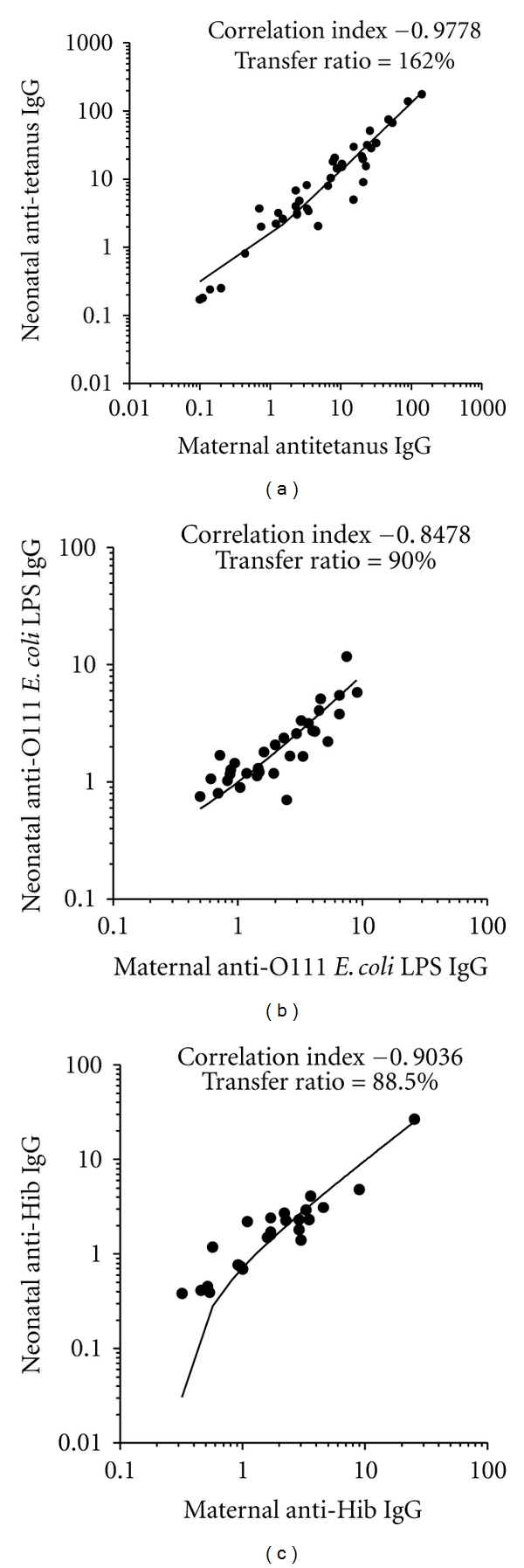
Correlation indexes and placental transfer ratios of maternal and term cord blood IgG levels reactive with tetanus toxoid, O111 LPS from enteropathogenic *E. coli* and Hib polysaccharide. Correlation indexes and placental transfer is higher to thymus-dependent antigens, as tetanus toxoid than to thymus-independent antigens type I and II, as LPS and polysaccharides, respectively.

The newborn IgG antibodies' levels usually correlate with maternal ones (Figure 2); however, the IgG binding to FcRn receptor can be saturated. Thus, the amount of IgG transmitted depends on the amount of cell surface receptors, because unbound IgG molecules are digested by lysosomal enzymes inside the vesicles [40]. This has been reported in several works performed in certain regions of Africa showing lower cord/maternal placental transfer ratios of total IgG, indicating that this limitation of active placental transfer of antibodies is related to the higher maternal IgG levels common in Africa [41–43]. It was reported by Michaux et al. [44] that total IgG concentrations in cord sera tend to be lower than in their mothers when total IgG levels in maternal serum reached 15 g/L. This is in agreement with other works that have demonstrated significant negative correlations between maternal levels of IgG and placental transfer ratios to the neonate for both total IgG and, interestingly, IgG specific to measles, LPS and other antigens [41, 45, 46].

Since the 1970s, Mäntyjärvi et al. [47] have demonstrated that neonatal anti-influenza A2 IgG levels on average tends to exceed that of the mother if the maternal level is low or normal. When the mother has a high content of total IgG or of a specific antibody, the neonatal value usually remained below the maternal one. This inverse relationship between the efficiency of placental transfer to the respective maternal level was also demonstrated for herpes simplex virus, tetanus toxoid, streptolysin O, and *S. pneumoniae *[48]. This is an interesting observation, because it is known that placental transport is mediated by the interaction between the Fc portion of IgG and the FcRn receptor, in which the Fab portion of this immunoglobulin is not involved. However, this phenomenon suggests an involvement of antigenic specificity of the antibody for this transport, but further studies are needed to investigate the mechanism involved.

## 4. IgG Transport Depends on the Subclass

**Figure 3:**
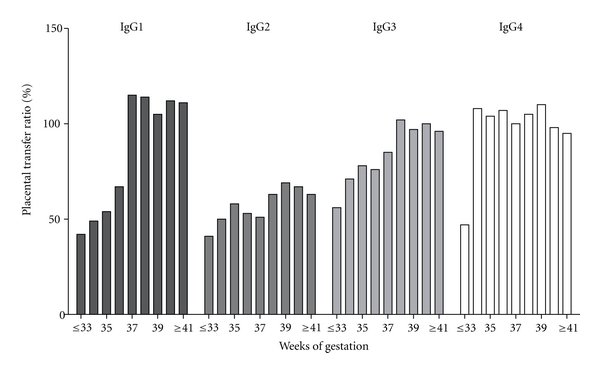
Percentages of placental transfer ratios of IgG subclasses delivered to preterm and term newborns in different gestational weeks. IgG1 and IgG3 transfer ratios rose with increasing gestational age, with IgG1 showing a peak transfer ratio at 37 weeks of pregnancy. IgG2 transfer ratios are always lower than the other IgG subclasses [52].

It is not clear why some antibody specificities exhibit different transfer impairments in different studies [49]. A plausible explanation may lie in variation in the IgG subclass responses to different antigens and the different affinities of these subclasses to the IgG-transporting FcRn receptors [50, 51]. Preferential transport occurs for IgG1, followed by IgG4, IgG3, and IgG2, for which the FcRn receptors have the lowest affinity [52] (Figure 3). This has been clearly demonstrated in studies on the transfer pattern of different types of specific IgG antibodies showing peculiarities in this transmission. IgG1 and IgG3 are transferred more efficiently across the placenta than IgG2. Furthermore, the transfer of antibodies against viral proteins and antitoxins of the IgG1 subclass occurs more readily. However, antibodies against encapsulated bacteria (*Haemophilus influenzae, Neisseria meningitidis, and Streptococcus pneumoniae*) in which IgG2 prevails, at least after natural exposure, are transferred less efficiently [53, 54], and an effective transplacental transmission of IgG antibodies reactive with LPS involving the IgG1 and IgG2 subclasses was confirmed in our previous studies [55].

In addition, it has been demonstrated that in term neonates with a low birth weight, all IgG subclasses were transferred with reduced efficiency, but IgG1 and IgG2 subclasses were transferred with significantly less efficiency than IgG3 and IgG4. These results demonstrate that low birth weight is associated with impaired placental transfer of IgG1 and IgG2 subclasses.

Overall, at term, IgG in cord blood has a good correlation with maternal levels, and placental transfer is systematically higher to thymus-dependent antigens (proteins), as tetanus toxoid than to thymus-independent antigens, both type I and II, as LPS and polysaccharides, respectively [56] (Figure 2).

## 5. IgG Transport Depends on Gestational Age

IgG transfer from mother to fetus begins as early as 13 weeks of gestation, and transport happens in a linear fashion as the pregnancy progresses, with the largest amount transferred in the third trimester [39]. Malek and colleagues [57] demonstrated a continuous rise in IgG levels in the fetal circulation between 17 and 41 weeks of gestation. Fetal IgG concentrations were only 5%–10% of the maternal levels at weeks 17–22 but reached 50% of the maternal concentrations at weeks 28–32. The majority of IgG is acquired by the fetus during the last 4 weeks of pregnancy, and fetal IgG concentrations usually exceed maternal ones by 20%–30% at full term [39]. Interestingly, a sharp increase in cord blood levels occurs after the 36th week of gestation.

**Figure 4:**
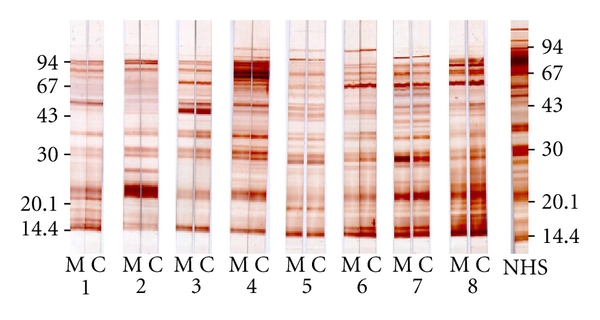
Immunoblotting of anti-EHEC O157:H7 IgG antibodies in the paired maternal and cord samples. Bacterial proteins were separated by 12.5% SDS-PAGE. Paired maternal and cord serum samples are identified numerically. M: maternal serum; C: cord serum; NHS: pool of healthy adult serum samples (normal human serum). The immunoblots were developed with antihuman IgG conjugate. Molecular weight standards are on the left for samples 1–8 and on the right for the pool of normal human serum. It was observed that there is almost complete identity between the antigens recognized by maternal and umbilical cord sera [58].

At term, dependent on the immunological experience of the mother, placental transfer allows the newborn to acquire different specificities of IgG antibodies, resulting in an identical recognition pattern of antigens between the mother and her offspring. As shown in Figure 4, an immunoblotting assay demonstrates identical patterns of enterohemorrhagic *E. coli* (EHEC) antigen recognition between paired mother and term cord sera, thus confirming abundant transfer of the maternal antibody repertoire to the newborn at least for protein antigens.

Maternal age, weight, parity, and type of delivery do not influence placental antibody transfer [59]. 

**Figure 5:**
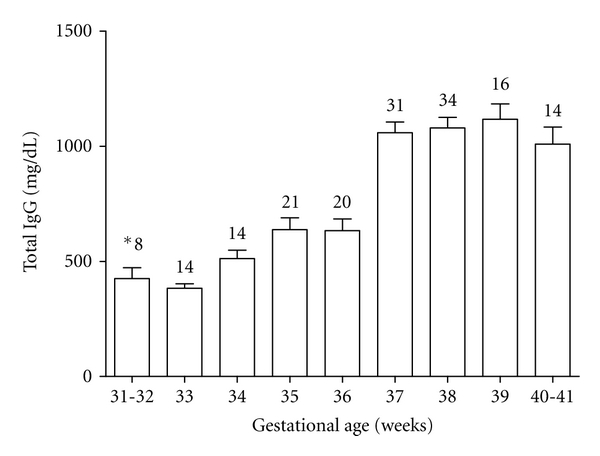
Total IgG concentrations in cord serum samples from newborns in different gestational weeks [46, 60]. *Number of samples in each period.

Total IgG concentrations in newborns, therefore, are directly related to length of gestation, and infants born at less than 33 weeks of gestation have substantially lower IgG levels than full-term babies (Figure 5). As the expression of FcRn receptor is dependent on gestational age and seems to be more highly expressed in the third trimester of human pregnancy, a reduced placental transfer of antibodies is observed at early stages of gestation. This fact results in a reduced transfer of IgG subclasses, especially IgG1 and IgG2, in preterm compared with full-term babies [56]. 

Accordingly, in a recent study, van den Berg et al. [54] found significantly lower transplacental transmission of IgG in preterm infants (<32 weeks) than in full-term infants for antibodies against diphtheria, tetanus, pertussis, *Haemophilus influenza* type b (Hib), and *Neisseria meningitides* serogroup C. In agreement with these data, Silveira Lessa et al. [46] evaluated the placental transfer ratios of IgG antibodies reactive with *Klebsiella*, *Pseudomonas,* and *E. coli* O111, O26, and O6 lipopolysaccharides and showed lower anti-LPS IgG transfer ratios in preterm groups (<33 weeks and >33 weeks) as compared with term ones (>37 weeks).

## 6. The Influence of Maternal Immunization

A main focus of the study of IgG transport across the placenta is maternal vaccination. Currently, several routine vaccines are recommended for pregnant women, such as tetanus toxoid vaccine and inactivated influenza virus vaccine. Others are used in special circumstances, including polysaccharide vaccines, such as pneumococcal polysaccharide vaccine and meningococcal polysaccharide vaccine, and inactivated viral vaccines, such as hepatitis A and B, rabies virus, or inactivated poliovirus vaccines [61]. All of these vaccines are given to protect women from serious diseases during pregnancy and the postpartum period while potentially providing benefits to the fetus and neonate due to placental transmission of those maternal antibodies.

However, many factors may limit the placental transfer efficacy after maternal vaccination, such as the timing between immunization at the pregnancy and delivery, the gestational age of the fetus at birth, total maternal IgG levels and the maternal vaccine-specific IgG and IgG subclasses concentrations [62, 63].

Several randomized studies have been conducted aiming to study the effectiveness of maternal vaccination, targeting important pathogens in early childhood [64, 65]. Prospective studies have demonstrated higher cord antibody levels to influenza in babies born to mothers immunized during pregnancy [66]. Infants of vaccinated mothers were 45%–48% less likely to have influenza hospitalizations than infants of unvaccinated mothers [67]. Maternal influenza vaccination effectiveness for both mother and newborn was also demonstrated in developing countries [68, 69].

Recently, it was showed that Tdap (tetanus and diphtheria toxoids and acellular pertussis antigens) vaccination in pregnancy was safe and significantly increased antibody titers against those antigens. These data reinforced that maternal Tdap vaccination in the second trimester may prevent neonatal pertussis disease in the first 5-6 months of life until infants receive active vaccinations with Tdap at 2, 4, and 6 months of age and establish active immunity [70].

In clinical studies, several factors can affect the transport of antibodies specific to vaccine antigens, particularly the type of vaccine administered. Vaccines with the ability to induce higher maternal levels of IgG and specifically IgG1, such as Hib polysaccharide- (PRP-) conjugate vaccines result in increased concentrations of IgG1 delivered to the fetus [61] and significantly more PRP-specific IgG antibodies during at least the first 2 months of life in diverse populations [71–75]. The same was observed for type III capsular polysaccharide of Group B Streptococcus (GBS) conjugated to tetanus toxoid vaccine, demonstrating an efficient transport of GBS-specific IgG antibodies to the neonate [76].

Reports on maternal-fetal transfer of antibodies against the capsular polysaccharides of *S. pneumoniae* have demonstrated that even term infants generally receive only a fraction (50%–85%) of either naturally acquired [77] or polysaccharide vaccine-induced antibodies from their mothers [63, 78–82]. Although higher IgG antibody levels are found in offspring of immunized compared to unimmunized women, these titers are not maintained for a long time after birth, they likely increase protection from invasive pneumococcal disease until around 120 days after birth, when disease rates are very high [78]. In contrast to the polysaccharide vaccine, maternal immunization schedules including a conjugate pneumococcal vaccine have the potential advantage of stimulating a larger quantity of antibodies of the IgG1 rather than the IgG2 subclass [83].

Another point that merits discussion is that higher doses of passively acquired antibodies may suppress antibody responses to active immunization in early infancy. Several studies have also reported that maternal antibodies can inhibit infant responses to measles, tetanus, whole cell pertussis, and Hib vaccines; this effect varies considerably between different vaccines and studies [84–87]. Regarding toxoids, it was observed that infants who had considerable levels of pre-existing antibodies exhibited lower responses after active immunization to diphtheria toxoid after the second dose, but after 12 months of life, antibody titers do not differ between those infants whose mothers had low titers. For the conjugate PRP-T vaccine, the anti-Hib antibody response was not affected by high maternal antitoxin titers; however, the infants' response to tetanus toxoid was dampened by these high titers. Despite this, all infants achieved protective levels of tetanus antitoxin-IgG after the booster dose with PRP-T. Regarding polysaccharide vaccines, studies have shown no difference in immune response of infants whose mothers received the vaccine or not during pregnancy when they are given the doses at 6–8 months of life. This observation was made with both meningococcal polysaccharide and Hib vaccines [88].

The mechanisms through which maternal antibodies inhibit infant responses to vaccination are not fully understood. However, some plausible explanations are as follows: (i) neutralization of live viral vaccines, (ii) vaccine antigen immune complexes inhibiting infant B cell activation mediated by Fc*γ*RIIb receptor, (iii) effective elimination of vaccine antigen coated with maternal IgG antibodies via Fc-dependent phagocytosis, and (iv) vaccine antigenic epitopes being masked or hidden by maternal antibodies, preventing binding by infant B cells [84, 89]. Although persistence of maternal antibodies may limit infant antibody responses, induction of T-cell responses remain largely unaffected by these passively transferred antibodies, because the administration of repeated vaccine doses, as routinely performed for diphtheria-tetanus-pertussis-polio and Hib vaccines, is often sufficient to overcome inhibition by maternal antibodies [90].

## 7. Placental Transport of IgG in Infectious Diseases

It is well known that antibody transport during pregnancy can be affected by a number of factors and clinical conditions, including placental abnormalities, total IgG concentration in maternal blood, the gestational age of the fetus at birth, and maternal pathologies, such as hypergammaglobulinemia, HIV infection, and placental malaria [91–93]. In addition, preterm labors and intrauterine growth retardation are associated with a number of pathologies, such as chronic hypertensive disease or hypertensive disease during pregnancy, preeclampsia, gestational diabetes, and infections whose influence in maternal antibody levels is still unknown [94].

In cases of maternal HIV infection or placental injuries, like malaria, a great decrease in antibody transfer has been reported [48, 75, 95–97]. A multivariate regression analysis study determined that placental malaria or maternal HIV infection, independent of maternal hypergammaglobulinemia, are conditions that affect placental transfer of antibodies, and if the mother also has high IgG serum levels, placental transfer is even more impaired [92].

It has been recently demonstrated that HIV-exposed but uninfected infants have reduced transplacental transfer of Hib-, pertussis-, pneumococcus-, and tetanus-specific antibodies than their non-HIV exposed peers. These findings were consistent with two other studies in HIV-infected women from Kenya, indicating that maternal HIV is associated with lower tetanus and measles-specific antibodies in cord blood and also with reduced placental antibody transfer [98, 99]. However, although prenatal HIV exposure was associated with lower specific antibody levels in exposed uninfected infants compared with unexposed infants at birth, after 16 weeks of life, robust and significantly higher antibody responses to pertussis and pneumococcus following routine vaccination were observed in the group of exposed uninfected infants compared with control infants. Therefore, HIV exposure is associated with a greater change in antibody levels between birth and 16 weeks [100].

## 8. Placental Transfer in Mothers with Primary Immunodeficiencies

Women with humoral deficiencies are dependent on exogenous administration of lgG to prevent recurrent infections with possible severe morbidity and even mortality. In addition, in the absence of the intravenous immunoglobulin (IVIG) therapy, their fetuses may also have an increased risk of infection during intrauterine life and during the first few months after birth because of reduced transplacental transfer of immunoglobulins from those mothers to their offspring [101].

Common variable immunodeficiency (CVID) is not an extremely rare disorder, and currently, many patients reach childbearing age in reasonably good health and become pregnant. CVID represents a heterogeneous group of immunologic disorders, characterized by reduced serum immunoglobulin levels and impaired antibody responses, with variable T cell numbers and function [102]. Its genetic heterogeneity has been studied in the last few years, with the identification of underlying defects in the following genes: ICOS (inducible costimulator), BAFF-R (B-cell-activating factor receptor), TACI (transmembrane activator and calcium-modulator and cyclophilin ligand interactor), CD19, and, more recently, CD20 and CD81 deficiencies [103].

There are only a few reports on total immunoglobulin placental transfer in those cases [104–106], but it was recently shown that CVID mothers under IVIG therapy efficiently transferred exogenous IgG through the placenta in similar patterns as endogenous immunoglobulins, as demonstrated by the following: (i) cord blood IgG levels in term babies were even greater than in the mothers, (ii) a preferential transfer of IgG1, IgG3 and IgG4 compared with IgG2, (iii) antiprotein IgG antibody levels equivalent to or higher than maternal ones in cord serum and good transfer of antipolysaccharide IgG antibodies, and (iv) similar anti-*S. pneumoniae* avidity indexes between mothers and their respective neonates (Table 1) [107]. Thus, CVID patients must be informed about the relevance of regular IVIG administration during pregnancy not only for their own health but also for the immunity of their immature offspring.

## 9. Placental Transfer in Mothers with Autoimmune Diseases

There are circumstances in which placental transmission of antibodies is detrimental to the neonate. Neonatal lupus erythematosus (NLE) is a rare disease considered to be the exemplary prototypic model of passively acquired systemic autoimmune disease [108]. Maternal IgG autoantibodies against Ro/SSA and/or La/SSB or, less commonly, to U1-ribonucleoprotein (U1-RNP), are transported through the placenta and harm the fetus by causing injury to the skin (cutaneous rash). One of the strongest clinical associations is the development of congenital heart block, which is most often of third-degree severity in a structurally normal heart. This abnormality is an alarming prospect facing 2% of mothers with these autoantibodies [109]. The risk of having a second baby with NLE among women who have already had a baby with NLE increases to 15% [110].

Sera of patients with autoimmune disorders contain an active idiotypic-anti-idiotypic network, which can also be induced in experimental animals following immunization with B-cell epitopes of autoantigens. It has been shown that sera of pregnant women with anti-La/SSB autoantibodies who carry a healthy baby have significantly higher levels of anti-idiotypic antibodies to anti-La/SSB, suggesting that these may serve as protective antibodies for the development of congenital heart block [111]. Therefore, the presence of anti-idiotypic antibodies to autoantibodies against La/SSB may protect the fetus by blocking pathogenic maternal autoantibodies. 

The transference of autoantibodies was also reported in neonatal pemphigus, which is characterized as a rare transitory autoimmune blistering disease caused by transfer of maternal IgG autoantibodies specific for desmoglein 3 to the neonate when the mother is affected with pemphigus [112]. This disease is clinically characterized by transient flaccid blisters and erosions on the skin and rarely the mucosa. However, by 3 months, IgG antidesmoglein levels in the neonate are within normal limits [113]. Transient neonatal autoimmune diseases have also been reported for myasthenia gravis and antiphospholipid syndrome, and recently, a case was reported of a newborn with transient epidermolysis bullosa acquisita, a chronic, autoimmune bullous dermatosis due to the passive transfer of maternal autoantibodies against the noncollagenous terminus of the *α* chain of type VII collagen [114–116].

In autoimmune diseases in which pathogenic or excess IgG antibodies are the etiological agents, such as myasthenia gravis, bullous pemphigoid, idiopathic thrombocytopenic purpura (ITP), and systemic lupus erythematosus (SLE), it is sometimes advantageous to reduce endogenous serum IgG levels by interfering with FcRn function. One possible way to interfere with the function of FcRn is to overload it with “innocuous” IgG. As FcRn functions as the IgG homeostatic receptor, the level of FcRn expression determines the serum concentration of IgG. Administering large quantities of exogenous IgG raises the serum concentration above this equilibrium set point and saturates FcRn [117]. As a result, the excess IgG that does not bind to FcRn enters the degradative pathway. This results in a shortening of the serum IgG half-life. High-dose IVIG treatment is thought to exert an immunomodulatory effect by numerous mechanisms, including engagement of the inhibitory Fc*γ*RIIb receptor [118] and by FcRn saturation [117].

In mouse models of bullous pemphigoid, ITP and autoimmune arthritis, IVIG treatment results in the dilution of pathogenic antibodies to levels beneath the disease-causing threshold [30, 119, 120]. The fact that a therapeutic effect for IVIG is maintained in Fc*γ*RIIb-deficient mice and is attenuated in FcRn-deficient mice is strong evidence that an important mechanism of action of IVIG is its ability to compromise FcRn function [30, 121]. This approach provides a valuable tool to prevent neonatal autoimmune disease by exploiting the saturation of FcRn by high doses of IVIG [122–125].

Finally, one interesting point that has been well explored in murine models but not yet in humans is that placental-derived IgG antibodies exert long-life immunoregulatory functions, including imprinting the fetal immune network [126]. Thus, by crossing the placenta, maternal IgG, in addition to providing anti-infectious protection to the infant, could have other active immunoregulatory long-term effects. This mechanism of transplacental antibodies transfer could also be involved in the recognition of allergens and priming of small populations of allergen-specific T cells in the newborn during intrauterine life, which could represent a normal stimulatory signal [127]. 

## 10. Conclusions and Perspectives

The maternal IgG antibody transfer varies as a result of total and specific maternal IgG levels, IgG subclass (and thus, the nature of antigen), gestational age, and placental integrity. Knowledge of the features of placental transmission of IgG antibodies is crucial to exploit and manipulate this mechanism to benefit the newborn. The finding that mothers respond well to vaccination and are able to transfer their entire antibody repertoire to their infants is encouraging, raising the possibility of providing protection until the time when the infant is vaccinated. Overall, the employment of IVIG therapy promises to be an area of active research with applications in mothers with primary immunodeficiencies to promote maternal and newborn protection against infections and in the treatment of various antibody-mediated autoimmune diseases, modulating transfer of harmful autoantibodies.

## Figures and Tables

**Table 1:** Serum immunoglobulin levels, specific antibody concentrations and avidity indexes in the maternal and cord serum and cord/maternal serum ratios for IgG antibodies from a mother with CVID.

	Maternal	Cord	Placental Transfer ratio (%)
Total Immunoglobulin Concentrations			

IgG (mg/dL)	473.0	912.0	190
IgM (mg/dL)	<6.0	12.0	–
IgA (mg/dL)	<3.0	<3.0	–
IgG1 (mg/dL)	362.0	752.0	210
IgG2 (mg/dL)	249.0	192.0	80
IgG3 (mg/dL)	10.0	20.0	200
IgG4 (mg/dL)	6.0	21.0	350

Specific IgG Antibodies Levels			

IgG anti-tetanus toxoid (UI/mL)	1.6	3.1	190

IgG anti-Hib PRP* (mg/L)	3.8	3.7	100

IgG anti-PS^§^1 (mg/L)/avidity (M)^+^	2.3/>3.0	2.6/>3.0	110
IgG anti-PS3 (mg/L)/avidity (M)	2.4/>3.0	2.8/>3.0	120
IgG anti-PS5 (mg/L)/avidity (M)	7.3/2.7	8.0/2.7	110
IgG anti-PS6 (mg/L)/avidity (M)	6.9/2.7	6.6/>3.0	100
IgG anti-PS9 (mg/L)/avidity (M)	4.4/2.9	4.7/>3.0	110
IgG anti-PS14 (mg/L)/avidity (M)	12.8/2.5	15.0/2.8	120

–IgM and IgA maternal/cord blood ratios were not performed;

*PRP—polyribosyl–ribitolphosphate polymers;

^§^Anti-PS—Anti-*Streptococcus pneumoniae* polysaccharide;

^+^(M) —Avidity index in molarity.
